# Recruitment of Latinx Older Adults With Parkinson Disease for a Remote Physical Activity Intervention Trial

**DOI:** 10.1093/geroni/igaf122.928

**Published:** 2025-12-31

**Authors:** Cristina Colón-Semenza

**Affiliations:** University of Connecticut, Storrs, Connecticut, United States

## Abstract

Recruitment for a completely remote, culturally and linguistically tailored, peer supported, physical activity intervention for older Hispanic/Latinx adults with Parkinson disease (PD) was evaluated. We employed both direct and indirect recruitment methods. Direct methods included direct contact by a study team member that was bilingual/bicultural (Spanish and English). Direct contact occurred via individualized telephone calls or emails from the study team (previous research participants), individualized letters from their treating neurologist (research team member), or research team member presentations in support group meetings. Indirect methods were also targeted at Hispanic/Latinx populations and available in Spanish and English but included general announcements or education that were presented to large audiences. This included recruitment flyer postings on social media, a news article within a university publication, organizational newsletters or listservs, large general community events in Latinx communities, shared with healthcare providers and within healthcare systems, and national webinars. Thirty-six individuals expressed interest in the study over a 6-month period (6 individuals/month). Fifty-six percent (20/36) were recruited through direct methods and 44% (16/36) through indirect methods. Twenty-nine individuals were screened, with 34.5 % consented. Of the 10 individuals that participated, 80% (8/10) were recruited through direct methods. The recruitment method that was most effective was direct contact by a research team member from within the target community. All 10 individuals that passed the screening and were consented were retained for the 12-week study duration. Direct methods, although labor intensive, resulted in greater numbers of underrepresented participants recruited and retained in this physical activity intervention.

